# Advancing brain tumor classification through MTAP model: an innovative approach in medical diagnostics

**DOI:** 10.1007/s11517-024-03064-5

**Published:** 2024-03-14

**Authors:** Cuneyt Ozdemir, Yahya Dogan

**Affiliations:** Computer Engineering, Engineering Faculty, Siirt University, Siirt, 56100 Turkey

**Keywords:** Brain tumor, ADASYN, Convolutional neural network, Pruning, Avg-TopK pooling

## Abstract

**Abstract:**

The early diagnosis of brain tumors is critical in the area of healthcare, owing to the potentially life-threatening repercussions unstable growths within the brain can pose to individuals. The accurate and early diagnosis of brain tumors enables prompt medical intervention. In this context, we have established a new model called MTAP to enable a highly accurate diagnosis of brain tumors. The MTAP model addresses dataset class imbalance by utilizing the ADASYN method, employs a network pruning technique to reduce unnecessary weights and nodes in the neural network, and incorporates Avg-TopK pooling method for enhanced feature extraction. The primary goal of our research is to enhance the accuracy of brain tumor type detection, a critical aspect of medical imaging and diagnostics. The MTAP model introduces a novel classification strategy for brain tumors, leveraging the strength of deep learning methods and novel model refinement techniques. Following comprehensive experimental studies and meticulous design, the MTAP model has achieved a state-of-the-art accuracy of 99.69%. Our findings indicate that the use of deep learning and innovative model refinement techniques shows promise in facilitating the early detection of brain tumors. Analysis of the model’s heat map revealed a notable focus on regions encompassing the parietal and temporal lobes.

**Graphical Abstract:**

Grad-CAM heat map visualization results
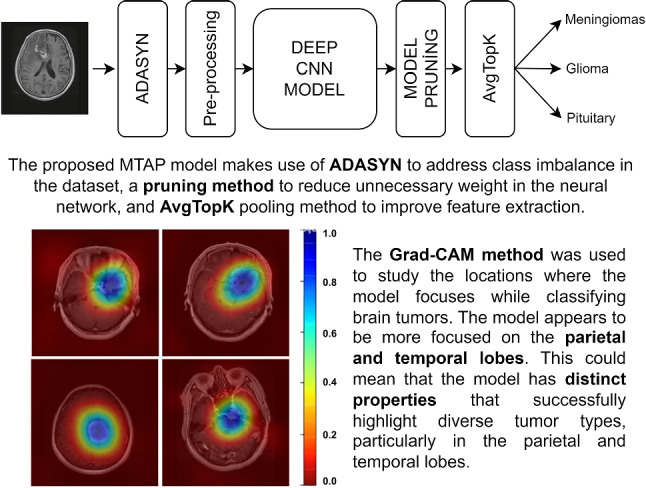

## Introduction

A brain tumor is a mass or abnormal growth of cells within the brain or the central spinal canal. This uncontrolled growth can arise from various cell types within the brain, including glial cells, neurons, and meningeal tissues. The development of brain tumors can result from genetic mutations, exposure to ionizing radiation, certain hereditary conditions, or environmental factors. Genetic mutations may disrupt the normal regulatory mechanisms that control cell growth and division, leading to the formation of a tumor [1]. The classification of brain tumors is intricate due to the diversity of cell types within the central nervous system and the varied pathological behaviors exhibited by these tumors. The World Health Organization (WHO) classification system stratifies brain tumors based on histological, genetic, and molecular characteristics, resulting in a classification that encompasses over 100 different types and subtypes of brain tumors [2].

Tumors in the brain are divided into two categories: malignant and benign. Malignant tumors, if not detected in their early stages, can lead to significant damage to brain tissue and can be fatal. Malignant tumors are classified into subtypes such as meningiomas, gliomas, and pituitary tumors. Meningiomas originate in the cells surrounding the brain and can present challenges for accurate diagnosis. Meningiomas are often slow-growing tumors that originate in the meninges, the protective layers surrounding the brain and spinal cord. They are typically benign but can cause problems due to their location and potential compression of surrounding brain tissue [1]. Glioma is a type of brain tumor that originates from the supportive cells in the brain, known as glial cells, and can be of low or high grade depending on the severity of the tumor [2]. Pituitary tumors, which develop within the pituitary gland located at the base of the brain and often referred to as the master gland, are typically non-cancerous growths. These tumors can cause overproduction or underproduction of hormones, resulting in a variety of hormonal imbalances and associated symptoms [3]. Given their ability to cause damage to brain tissue and cause a variety of side effects, brain tumors should be identified as soon as possible, whether malignant or benign [4].

Diagnosing brain tumors involves a multidisciplinary approach, integrating clinical assessments, neuroimaging, and histopathological analysis. Neuroimaging techniques such as magnetic resonance imaging (MRI), computed tomography (CT), and positron emission tomography (PET) scans play a critical role in visualizing and locating tumors within the brain. Histopathological examination of biopsy or surgical specimens provides detailed insights into the tumor’s cell type, grade, and molecular features [2]. Brain tumor diagnosis is critical for effective therapy and improved patient outcomes. Early detection allows for the rapid beginning of suitable therapeutic measures, potentially boosting treatment success and lowering morbidity associated with advanced-stage malignancies [5]. Early diagnosis also offers the possibility of less invasive surgical procedures and better preservation of neurological function.

Brain tumors should be identified quickly and accurately to develop effective treatment methods, with characteristics such as tumor stage, pathological kind, and degree being crucial in treatment technique selection. The literature highlights numerous studies on computer-assisted medical diagnosis, notably those that use convolutional neural networks (CNNs), which are deep learning approaches for extracting relevant information from medical images such as MRI scans. Precision identification of tumor size and location is critical for successful diagnosis in MRI, a task usually accomplished by skilled doctors visually reading the data. On the other hand, computer-aided systems necessitate collaboration with skilled medical practitioners to identify critical image components for optimal system results. CNN technology automates these processes, leading to enhanced efficiency

This work contributes by creating a clinical decision support system to help healthcare workers who lack knowledge in detecting brain tumors from MR images. Recently, research in this field extensively employs transfer learning models. However, models prepared for larger datasets encompass numerous parameters, leading to expensive hardware and time costs. To expedite model predictions, this study introduces a simplified and computationally efficient CNN model. In this context, the weights of the model were examined to enhance computational efficiency and expedite model training, leading to the removal of unnecessary layers. This process facilitated the development of a leaner and faster model. Furthermore, performance was enhanced by integrating the Avg-TopK pooling layer instead of traditional pooling layers. The proposed model demonstrated promising potential as an alternative in the field, achieving state-of-the-art results.

## Related works

In recent times, the application of deep learning approaches has significantly enhanced diagnostic performance in computer-aided medical diagnoses. Particularly, sophisticated methods for the diagnosis of brain tumors have contributed to the effectiveness in this domain, contributing to scientific advancement. These scientific studies stand out as a significant step in improving diagnostic accuracy and effectiveness, allowing for the effective integration of technological developments into the healthcare services paradigm. This section discusses innovative approaches in the diagnosis of brain tumors.

Deepak and Ameer [6] developed an automated system utilizing support vector machine (SVM) and CNN features for classifying brain tumors in MRI images. Their model achieved an overall classification accuracy of 95.82% using the Figshare dataset.

Paul et al. [7] used deep learning methods to classify brain images associated with meningiomas, gliomas, and pituitary tumors. This investigation utilized the same dataset, comprising 3064 T1-weighted contrast-enhanced MRI brain images derived from 233 patients. The research involved the design and implementation of two distinct neural network architectures: fully connected networks and CNNs. Notably, a comprehensive evaluation using a five-fold cross-validation technique revealed that general methodologies achieved superior performance with an accuracy rate of 91.43% compared to specific methods that required image dilation.

Abd-Ellah et al. [8] utilized CNNs to automate the diagnosis of brain tumors using magnetic resonance images. The primary objective of this investigation was to distinguish between images of brains with pathology (tumors) and those of healthy brains. To facilitate this diagnosis, a two-stage multi-model system was created. In the initial phase, preprocessing and judicious feature selection were executed by a CNN. Subsequently, an error-correcting output codes support vector machine (ECOC-SVM) was employed to perform a classification task. In this initial phase, three distinct models—AlexNet, VGG16, and VGG19—were utilized. AlexNet exhibited the most impressive performance, achieving a 99.55% accuracy rate. During the first phase, the BraTS (2013 dataset) was used to localize brain lesions, while images from the Reference Image Database to Evaluate Response (RIDER) neuro MRI database were used for performance evaluation.

Abiwinanda et al. [9] introduced an optimized CNN model for brain tumor classification. The design included two convolutional layers employing $3 \times 3$ kernels and a total of 32 filters. These layers were followed by an activation layer utilizing ReLU and a subsequent maximum pooling layer. A final layer, containing 64 neurons, culminated in the architectural composition. This design yielded an impressive classification accuracy of 84.19% in the diagnosis of brain cancers, demonstrating its efficiency and potential for medical image analysis.

Gumaei et al. [10] developed an innovative classification methodology for the analysis of brain tumors. Their approach harnessed a hybrid strategy, amalgamating advanced techniques for feature extraction with the finesse of a regularized extreme learning machine (RELM). They introduced a novel hybrid feature extraction technique christened PCA-NGIST, a fusion of principal component analysis (PCA), and the refined normalized Gabor filtered image statistical technique. The crux of their research lay in a meticulously conducted series of experiments and analyses, culminating in a discernible enhancement in performance. Notably, the classification accuracy witnessed a remarkable surge, ascending from 91.51% to an impressive 94.23%, as validated through a stringent evaluation employing a randomized technique.

Kaplan et al. [11] pioneered the use of two different feature extraction techniques, nLBP and $\alpha$LBP, for brain tumor classification. The classification study involved the use of various methodologies, including *K*-nearest neighbors (KNN), artificial neural networks (ANN), random forest (RF), A1DE, and linear discriminant analysis (LDA). Within the framework of this research and using the Figshare dataset, the KNN model combined with the LBPd=1 feature extraction technique achieved a 95.56% success rate in accurately classifying brain tumors.

Toğaçar et al. [12] employed a feed-forward CNN model named BrainMRNet. This model integrated a pioneering Hypercolumn technique, strategically engineered to enhance classification performance by discerning the most influential features from the input data while minimizing computational expenses. Upon conducting extensive experiments utilizing the Figshare dataset, they achieved a notable classification accuracy of 96.57%.

## Materials and methods

The Figshare dataset was used in this work, and cutting-edge results were obtained by merging several contemporary techniques into an established model. This section contains information about the dataset and the methods that were used.

### Dataset

**Fig. 1:**
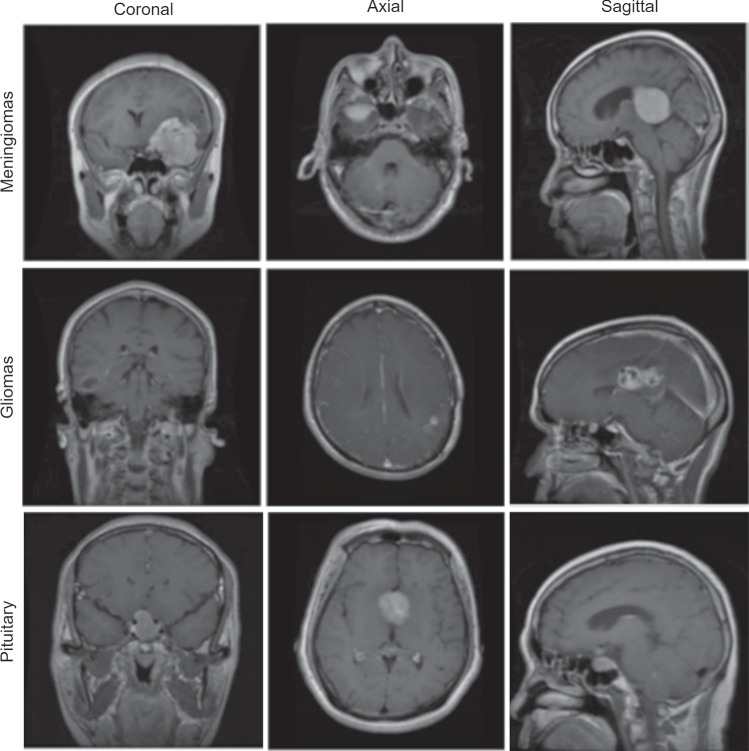
In the Figshare dataset, MRI images of meningiomas, gliomas, and pituitary tumors are presented through sagittal, coronal, and axial cross-sections [14]

The Figshare dataset, introduced by Cheng in 2015 and subsequently updated in 2017 [13], serves as a publicly accessible repository. This dataset includes images related to three prevalent types of brain tumors: meningiomas, gliomas, and pituitary tumors. The dataset comprises a total of 3064 images, distributed as 708 meningioma images, 1426 glioma images, and 930 pituitary tumor images. Table 1 provides the distribution of tumor types within the dataset. Furthermore, example images for three different types of brain tumors are provided in Fig. 1.

**Table 1:** Sample counts for each class in the Figshare dataset

Class name	Size	Percentage
Glioma	1426	46.5%
Meningiomas	708	23.1%
Pituitary	930	30.4%
Total	3064	

In experimental studies, the dataset was divided into two distinct categories: training and testing. The training set covers 85% of the dataset, consisting of 2604 images, while the remaining 15% (460 images) is allocated to the test set. To achieve robust results, the training dataset was partitioned into fivefolds, and a cross-validation process was applied, with fourfolds used for training and onefold for validation at each stage. The results presented in the tables indicate the average scores obtained through the cross-validation process.

### Method

In this study, a customized innovative CNN model has been proposed to distinguish between three different types of brain tumors using the Figshare dataset. The DenseNet201 architecture [15] has been utilized as the transfer learning model. The CNN model created for brain tumor classification consists of three main components: ADASYN, model pruning, and Avg-TopK.

#### ADASYN

is a potent tool in machine learning for mitigating class imbalance issues prevalent in datasets, particularly within classification tasks proposed by He et al. [16]. ADASYN focuses on scenarios where one class (the minority class) is significantly underrepresented compared to another (the majority class). This disparity can significantly skew model learning and performance. The essence of ADASYN lies in the generation of synthetic data, primarily aimed at augmenting the minority class. Unlike traditional oversampling techniques, ADASYN dynamically computes the number of synthetic samples needed for each minority class instance. This adaptability is crucial, as it enables the algorithm to generate more synthetic examples in regions with a lower class density, effectively resolving the difficult-to-learn minority examples.

#### Model pruning

Model pruning refers to the process of streamlining complex machine learning models by removing unnecessary parameters, connections, or weights. This technique aims to enhance model efficiency, reducing computational demands and memory requirements without compromising performance. By selectively eliminating redundant or less impactful parameters, pruning mitigates overfitting and enhances the model’s generalization capabilities. This approach contributes significantly to model optimization, allowing for leaner, more efficient neural networks that are better suited for deployment in resource-constrained environments.

#### Avg-TopK

Özdemir [17], a recent advancement in deep learning, provides a dynamic alternative to traditional pooling layers. Instead of using traditional pooling strategies, Avg-TopK dynamically adapts by calculating the average activations of the top *K* activations from a given layer. This adaptability allows the model to detect complex features and patterns that static pooling methods would otherwise miss. The utility of Avg-TopK lies in its ability to enhance feature representation. By considering the top activations, the model concentrates on the most prominent features within a layer, which may result in representations that are richer and more discriminative. This adaptability is especially useful in complex tasks such as the classification of medical images, where minute details significantly contribute to an accurate diagnosis.

**Fig. 2:**
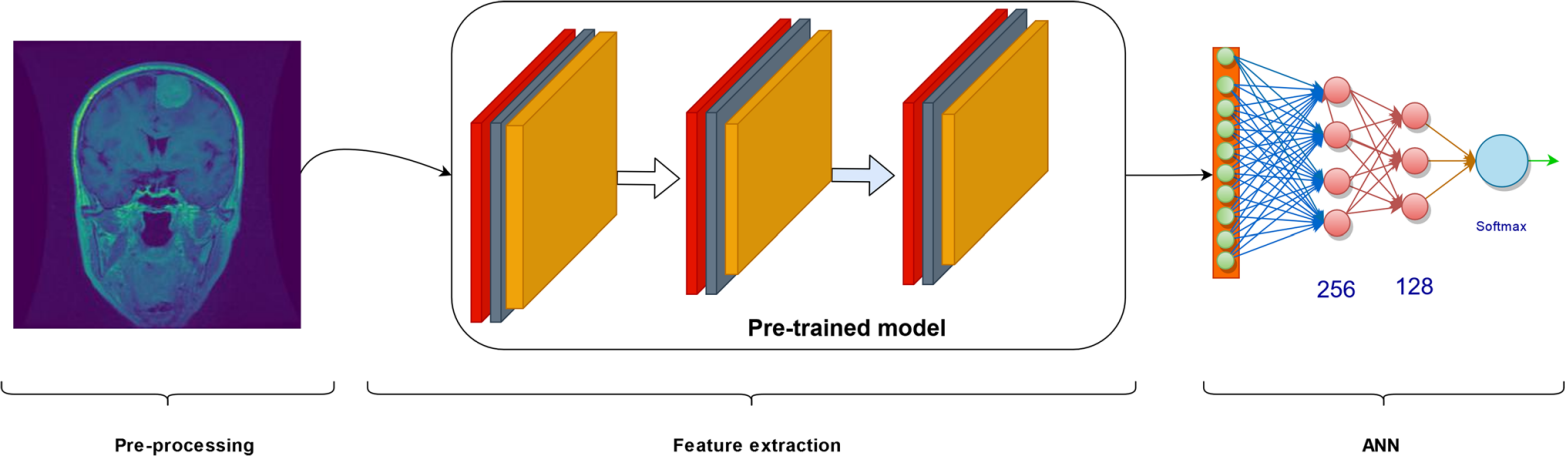
CNN architecture

Figure 2 depicts the architecture employed in experimental studies.

#### Preprocessing

In this initial stage, comprehensive preprocessing procedures were applied to all images within the dataset. An array of experimental investigations was conducted to assess the impact of preprocessing on the model’s overall performance, with specific attention given to optimizing parameter settings. A detailed account of these experimental studies is presented in the subsequent experimental studies section. The images can be accessed in the sagittal, coronal, and axial directions, with spatial resolutions of either $512 \times 512$ or $256 \times 256$. In experimental studies, all images were resized to a resolution of $256 \times 256$. Initially, a scaling process was employed on the dataset to resize all images uniformly to dimensions of $256 \times 256$. This resizing step is instrumental in expediting computational processing in deep learning methodologies, mitigating the computational burden arising from high-level mathematical operations, particularly in the context of varying image sizes and resolutions.

#### Feature extraction

In CNN models, features are automatically extracted, but training the entire network from scratch can require a large dataset and time. Therefore, transfer learning approaches pre-trained on large datasets, such as the ImageNet dataset, have been utilized. This model serves as an effective feature extractor, capturing relevant image features without the need to train the entire network from scratch. The initial layers of deep neural networks trained on general datasets often learn low-level features such as edges, textures, and shapes. Hence, being trained on datasets like ImageNet is particularly advantageous. In this phase, the pre-trained model acts as a powerful tool to understand and represent the intricate features present within the brain tumor images. This representation, or feature vector, obtained from the pre-trained model, encapsulates valuable information that is subsequently utilized to train the final classification model in the subsequent stage of the architecture, the ANN. The use of a pre-trained model considerably enhances the efficiency and effectiveness of the overall classification process for brain tumors, enhancing the model’s ability to discern intricate patterns and make accurate predictions.

#### Artificial neural network (ANN)

In this part, features obtained from the feature extraction part are integrated with the class layers. The ANN architecture consists of two fully connected layers with 256 and 128 neurons each, utilizing the ReLU activation function. The classification layer uses the Softmax activation function to generate a probability score ranging from 0 to 1, indicating class membership. Additionally, dropout regularization is employed after each dense layer to prevent overfitting. In this study, we used the Adam optimization algorithm with its default parameters as a reference. We utilized the ReduceLROnPlateau approach to dynamically adjust the learning rate. In this context, if there is no reduction in the validation loss for seven epochs of training, the learning rate is reduced by a factor of 0.8. This approach aids in decreasing the learning rate when the model’s performance does not improve over a certain period. It is often used to steadily improve the training process and obtain better results.

### Performance metrics

In this study, the efficacy of the proposed architecture was assessed through a comprehensive evaluation, comparing the obtained results with ground truth labels determined by domain experts. Key performance indicators utilized for this evaluation included the F1 score, recall, precision, and accuracy. These metrics provide a comprehensive assessment of the classification performance of the model by assessing aspects such as the true positive rate, the false positive rate, and the overall predictive accuracy.

#### Accuracy

Accuracy measures the proportion of precise predictions (true positives and true negatives) relative to the total number of predictions. It provides an overall assessment of the model’s correctness and is suitable for balanced datasets where the classes are evenly distributed.

#### Recall

Recall assesses the proportion of true positive predictions out of the total actual positives (true positives + false negatives). It gauges the model’s ability to correctly identify all relevant instances within the dataset.

#### Precision

Precision determines the proportion of true positives out of the total predicted positives (true positives + false positives). It reflects the accuracy of the positive predictions made by the model and is particularly important when minimizing false positives is crucial.

#### F1 score

The F1 score is a measure that combines both precision and recall into a single value. It is the harmonic mean of precision and recall and provides a balance between the two. A high F1 score indicates a good balance between precision and recall.

Table 2 provides performance metrics offering a detailed quantitative analysis of the model’s classification performance.

$$\begin{aligned} \begin{aligned} Recall&= \frac{TP}{TP + FN} \\ Precision&= \frac{TP}{TP + FP} \\ Accuracy&= \frac{TP + TN}{TN + TP + FP + FN} \\ F1 score&= \frac{2 \times Precision \times Recall}{Precision + Recall} \end{aligned} \end{aligned}$$

**Table 2:** Description of evaluation metrics

	Predicted (*P*)	Predicted (*N*)
Actual (P)	*TP*	*FN*
Actual (N)	*FP*	*TN*

### Training details

We use the Adam optimizer with a batch size of 32 and train all models from scratch for 20 epochs, setting the learning rate to 0.001 and epsilon to $1e - 07$. To dynamically adjust the learning rate based on validation loss, we implement the ReduceLROnPlateau strategy. This approach reduces the learning rate by a factor of 0.8 when the minimum validation loss stops improving after 7 epochs, ensuring that the best model is saved using model checkpointing based on validation loss. Throughout the training process, we use the categorical cross-entropy loss function to update the model weights. Table 3 shows the hyperparameters used in the training of the overall model.

**Table 3:** Hyperparameters of model

Batch size	32
Learning rate	0.001
Input size	256 × 256 × 3
Optimizer	Adam
Loss function	Categorical cross-entropy loss
Epoch	20
Epsilon	$1e-07$
Learning rate decay factor	0.8

**Fig. 3:**
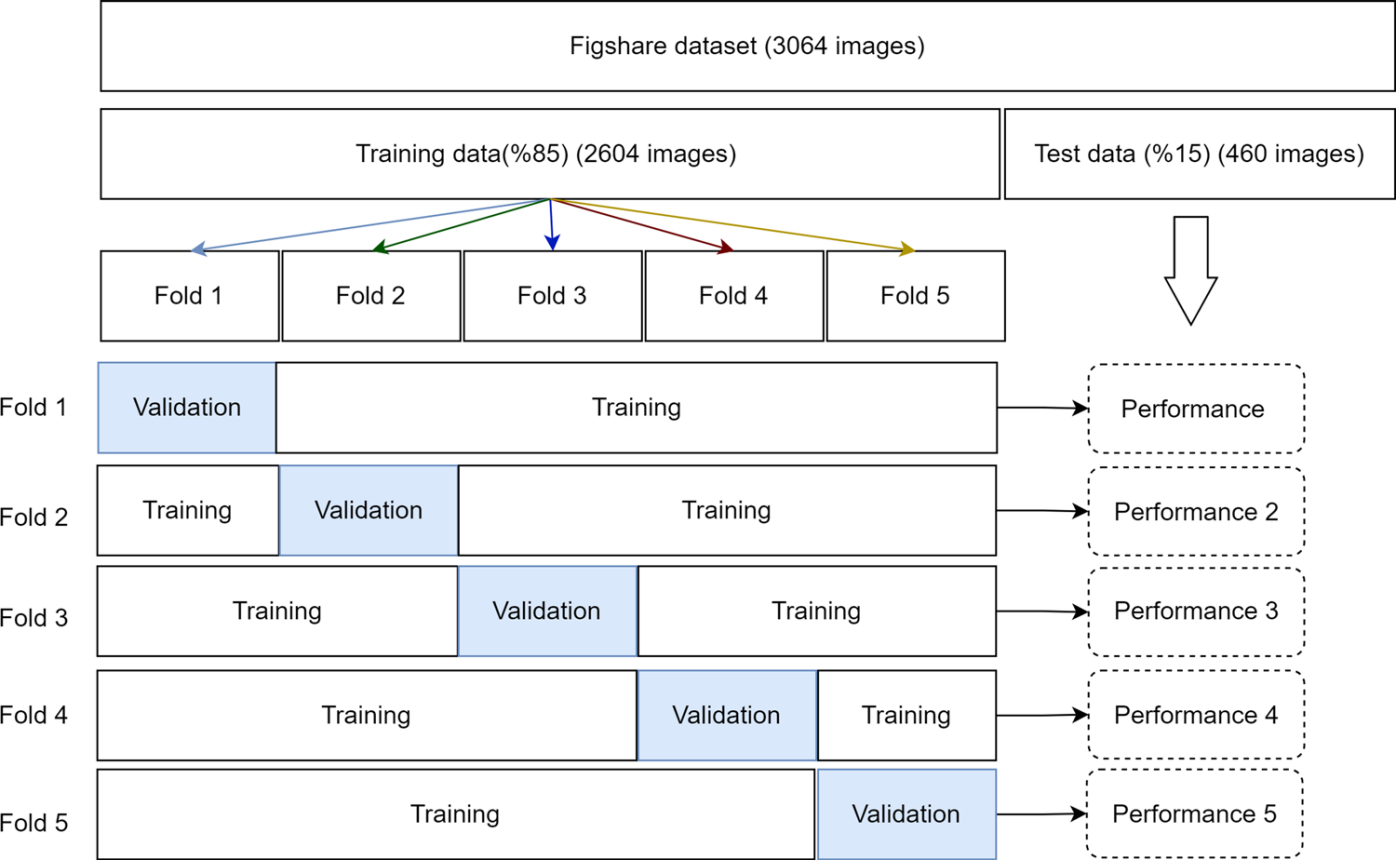
Cross-validation steps

## Experimental and results

In this study, a novel CNN model was developed specifically for the Figshare dataset, consisting of three different classes and a total of 3064 MR images. Due to the absence of a standardized method for splitting the dataset, we ensured model robustness through cross-validation. Initially, the dataset, consisting of 3064 images, was divided into an 85–15% split for training and testing, respectively. Following that, the training set was further partitioned into fivefolds. The model underwent iterative training using a fourfold training and onefold validation approach in each cross-validation step. Throughout these steps, the model was trained with the training set, hyperparameter optimized using the validation set, and finally tested with the test set. The visual representation of the cross-validation approach can be found in Fig. 3. We used the Eq. 1 to calculate the average performance of the model after cross-validation.

1 $$\begin{aligned} \mu = \frac{1}{5}\sum _{i=1}^{5} P_{i} \end{aligned}$$

Firstly, to determine the transfer learning model that exhibits the highest performance for the brain tumor classification problem, the performances of current transfer learning models were investigated. In this context, the performances of commonly referenced models in the literature, such as ResNet101V2, EfficientNetB3, ResNeXt-101, and DenseNet201, were examined. The results are presented in Table 4. Since the DenseNet201 model demonstrated the highest performance, subsequent experimental studies were conducted using this model as a reference.

Secondly, the performance of applying different preprocessing techniques to enhance model performance was investigated. In medical image classification studies, preprocessing techniques are commonly applied to enhance model performance and improve generalizability. The fundamental reasons for applying preprocessing to images are as follows:

1. Data diversity and generalization: Transformations such as rotation, zooming, and flipping enable the model to handle images with different poses, angles, and scales. This enhances the model’s generalization ability, making it better suited to real-world scenarios.
2. Overfitting reduction: Models that tend to focus on small details or noise in training data may fail on test data. Preprocessing helps the model focus on meaningful features and can reduce overfitting.
3. Dealing with data imbalance: Imbalances between classes are common in medical images. Preprocessing techniques can encourage more effective learning of underrepresented classes and alleviate imbalances.
4. Enhancing model robustness: Different image transformations make the model more resistant to noise, slight deformations, or minor changes. In the study, the impact of preprocessing applied to the dataset on performance was investigated.

**Table 4:** Model performances with and without preprocessing

	Accuracy	Precision	Recall	F1 score
ResNet101V2	88.12%	89.17%	88.12%	87.91%
EfficientNetB3	87.35%	88.39%	87.35%	87.15%
ResNeXt-101	88.37%	89.42%	88.36%	88.16%
DenseNet201	88.91%	89.97%	88.91%	88.70%

Experimental studies were conducted using the architecture shown in Fig. 2, with no fine-tuning applied, utilizing the DenseNet201 transfer learning model. A systematic approach was followed to determine preprocessing parameters. Initially, individual trials were conducted for each preprocessing parameter, followed by a comprehensive experiment covering all identified parameters. In all experiments, the data were resized to $256 \times 256$ dimensions, and normalization was applied. In this context, images were initially rotated by $\pm 15^{\circ }$, then zoomed in by $\pm 10\%$, and finally, performance was assessed by applying flipping. The comparative results obtained from the distinct models are presented in Table 5.

**Table 5:** Model performances with and without preprocessing

	Accuracy	Precision	Recall	F1 score
Without preprocessing	88.91%	89.97%	88.91%	88.70%
Rotation	89.51%	89.57%	89.51%	89.29%
Zooming	89.51%	89.58%	89.51%	89.29%
Flipping	89.89%	89.95%	89.89%	89.68%
With preprocessing	94.77%	95.26%	94.78%	94.81%

In the experimental studies section, a comparative analysis of model performance, with and without preprocessing, was presented (Table 5). The results demonstrate the impact of preprocessing methods on the classification accuracy of the proposed CNN model. Without preprocessing, the model achieved a test accuracy of 88.91%, precision of 89.97%, recall rate of 88.91%, and an F1 score of 88.70%. In contrast, when the dataset underwent preprocessing steps, the model’s performance significantly improved. The preprocessed model achieved a test accuracy of 94.77%, precision of 95.26%, recall rate of 94.78%, and an F1 score of 94.81%. These findings underscore the critical role of preprocessing techniques in enhancing the classification accuracy and overall performance of the CNN model. Applying preprocessing techniques to the dataset noticeably enhances the performance of the model. Additionally, concurrently implementing multiple preprocessing techniques provides a more pronounced contribution to boosting the model’s effectiveness. The presented results emphasize the importance of preprocessing steps in refining the model’s ability to discern brain tumor classes accurately, ultimately contributing to an efficient diagnostic process.

**Table 6:** Model pruning performance metrics

Model	Accuracy	Precision	Recall	F1 score
DenseConv4Block33	96.52%	96.64%	96.52%	96.46%

**Table 7:** Model pruning parameter sizes

Model	Total params	Trainable params	Non-trainable params
DenseNet201	18,847,043	18,617,987	229,056
DenseConv4Block33	11,833,283	11,701,187	132,096

Continuing with the study, a model pruning process was implemented with the aim of reducing model complexity and computational costs while optimizing model performance. The primary objective of model pruning is to eliminate layers that make insignificant contributions to the overall performance of the model. In the model pruning methodology, initially, the model is trained from scratch. Following that, the trained model is used to make predictions on images, and standard deviation and mean values for the weight parameters in each layer are produced during this prediction phase. The model then assesses these statistical values against a manually determined threshold to identify layers for removal. The threshold value is computed by taking into account layers with significant changes in average and standard deviation values. In future steps, this criterion led to the systematic trimming of layers. During this process, layers below the threshold value are removed, resulting in the derivation of a new model. This method has optimized the model architecture by eliminating layers with the least impact, thereby creating a model that enhances the overall efficiency and computational performance of the system.

**Table 8:** Performance results obtained from model pruning and ADASYN integration

Model	Accuracy	Precision	Recall	F1 score
DenseConv4Block33+ADASYN	99.53%	99.53%	99.53%	99.53%
DenseNet201+ADASYN	99.37%	99.37%	99.37%	99.37%

**Table 9:** Performance results obtained from model pruning, ADASYN, and Avg-TopK integration

Model	Test accuracy	Precision score	Recall score	F1 score
MTAP	99.69%	99.69%	99.69%	99.69%

In the DenseNet201 model, standard deviation and mean values were examined for each layer. Through manual observation, a plateau in model learning was observed beyond the layer conv4_block33_concat. Starting from this point, layers were removed from the model, resulting in a shallower model. The training process, considering the model obtained through model pruning and the best-performing preprocessing experimental setting, resulted in an observed increase in model performance. Table 6 presents the results of this experimental investigation.

As seen in Table 6, the DenseConv4Block33 model demonstrated, achieving a remarkable test accuracy of 96.52% with high precision, recall, and F1 scores, demonstrating the model’s in classifying brain tumor data. Table 7 provides the capacities of the pruned DenseConv4Block33 model compared to the DenseNet201 model. The DenseConv4Block33 model, which underwent pruning, experienced a reduction of approximately 37.20% in parameter size. This reduction signifies improved efficiency of the model, providing a lighter and faster architecture crucial for rapid inference and resource optimization in real-world clinical applications. Pruning process not only significantly reduced the model capacity but also led to an increase in classification performance.

In datasets obtained in the field of healthcare, the difficulty of acquiring data for specific classes often leads to the emergence of the class imbalance problem. Class imbalance refers to the unequal distribution of examples among different classes in a dataset. This situation can pose challenges in evaluating model performance. Class imbalance is a common issue in machine learning, particularly in classification tasks, and requires preventive measures. This imbalance can introduce bias into the model, causing it to prefer the majority class and consequently impeding the accurate identification of minority classes. Such bias can have significant implications in the context of medical diagnoses. For instance, in the classification of brain cancers, the accurate recognition of each tumor type is crucial for designing appropriate treatment approaches. An inconsistent dataset may result in the successful identification of the most common tumor types but may fail to detect the less prevalent ones. In the Figshare dataset under consideration, there is a significant disparity in the sample sizes among three distinct brain tumor types: meningiomas, gliomas, and pituitary tumors. The dataset comprises 708 meningioma images, 1426 glioma images, and 930 pituitary tumor images. Notably, the meningioma class constitutes approximately half the number of images in the glioma class. To mitigate the adverse effects of class imbalance and ensure a balanced learning process, the ADASYN (adaptive synthetic sampling method) [16] method was employed.

**Fig. 4:**
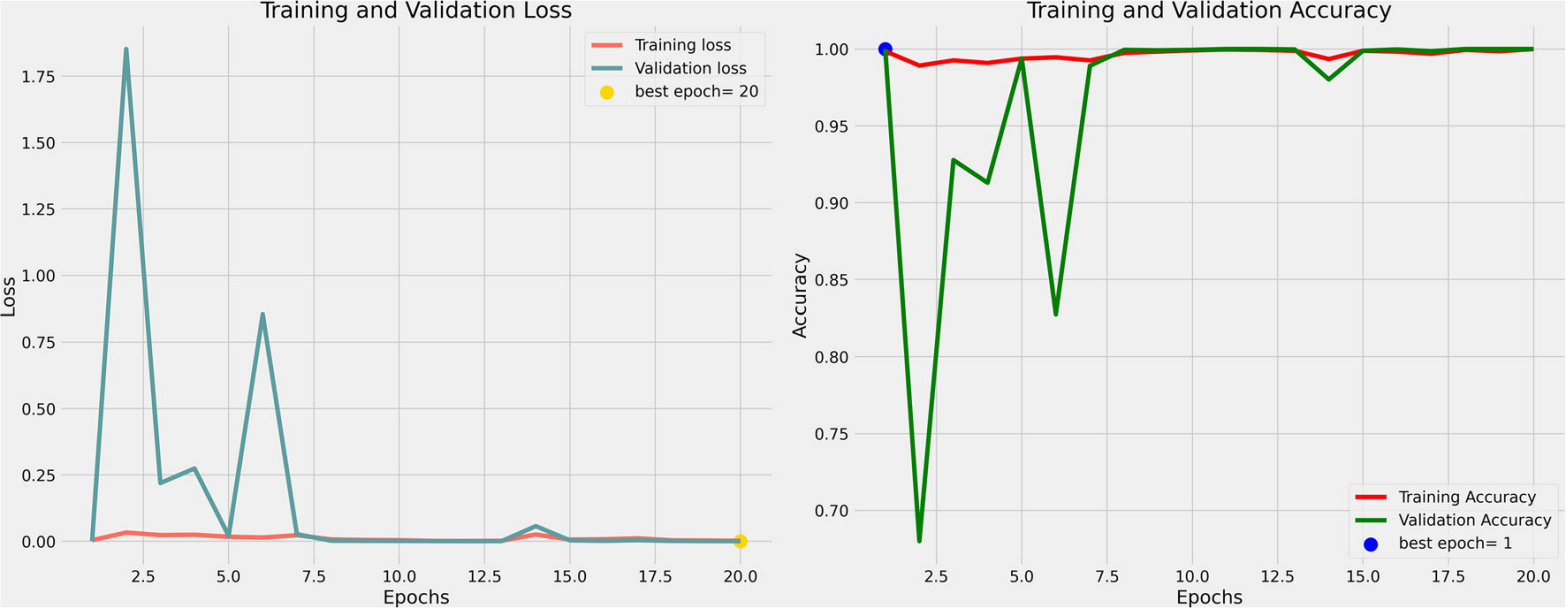
Loss and accuracy of the MTAP model

**Fig. 5:**
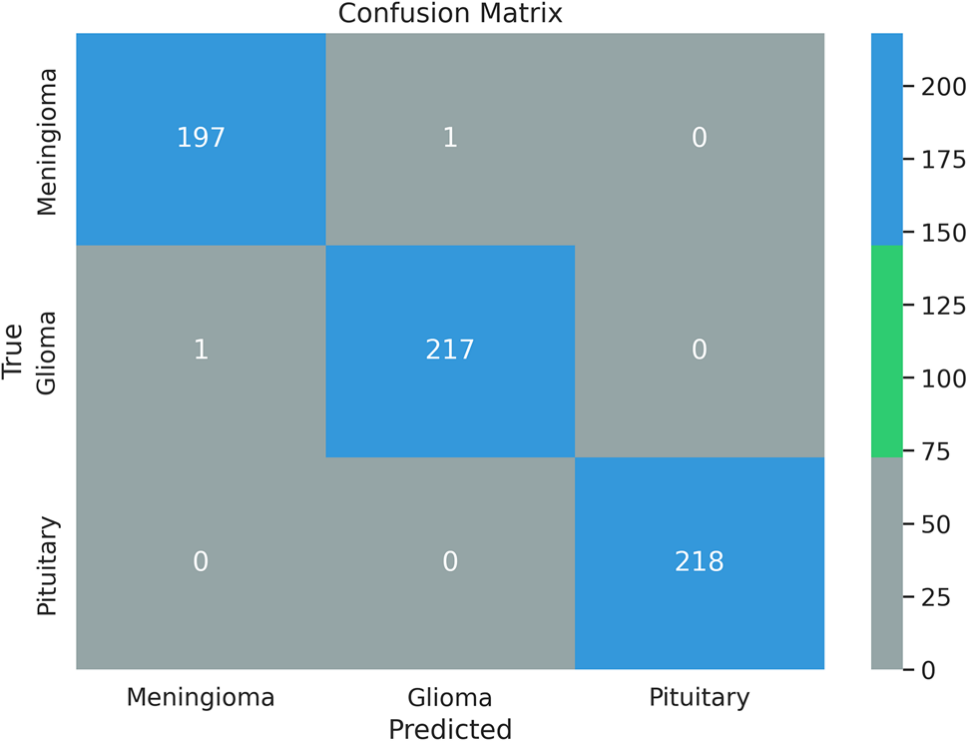
Confusion matrix of the MTAP model

The results obtained when addressing the data imbalance problem using the ADASYN method are presented in Table 8. These findings demonstrate the significance of correcting class imbalance and the positive impact of obtaining a balanced dataset on model performance and medical diagnostic potential. The accuracy of the model, obtained through the pruning process with the ADASYN method, increased from 96.52 to 99.53%. These results demonstrate that combining model pruning and the ADASYN method significantly contributes to the classification performance of the model.

**Fig. 6:**
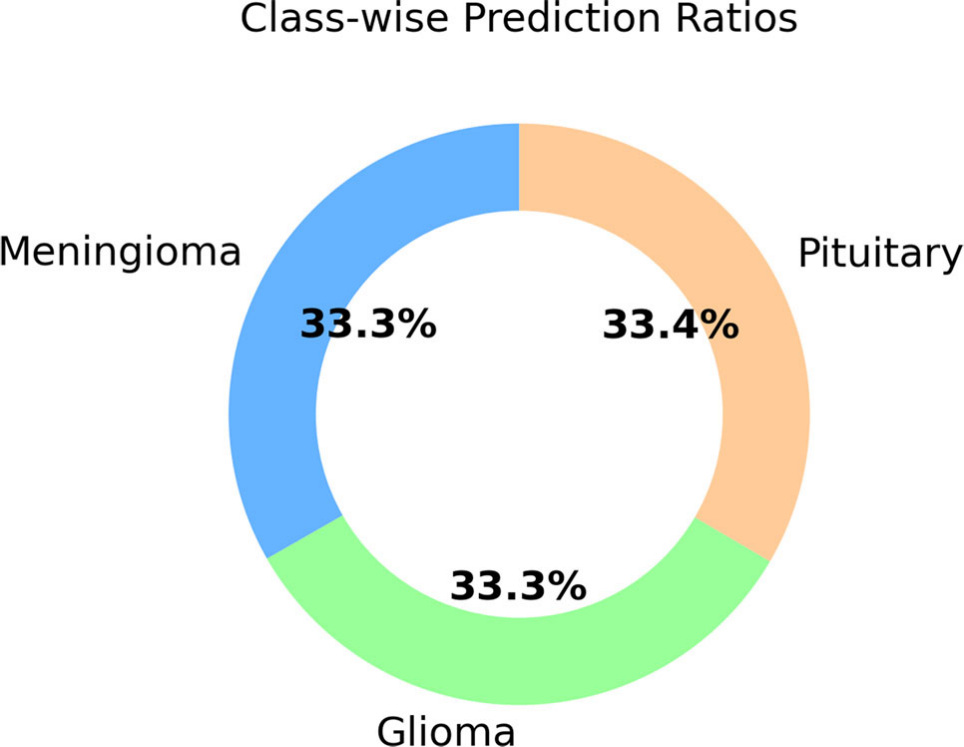
Class-wise prediction ratios in the model

The final alteration we applied in our efforts to increase model performance was the use of the Avg-TopK method. The local pooling layers in the DenseConv4Block33 model were replaced with Avg-TopK. Instead of depending on the single greatest intensity value, this method computes the average of the top *K* intensity values within the filter region. Table 9 details the results of this adjustment, including accuracy and loss metrics. As the study develops, the model created by combining model pruning, ADASYN, and Avg-TopK methods to improve performance is referred to as MTAP. Figure 4 depicts the accuracy and loss graph for the MTAP model, while Fig. 5 depicts the MTAP model’s confusion matrix.

**Table 10:** Methods and results used in studies with Figshare dataset

References	Feature extraction	Model	Accuracy (%)
Cheng et al. [18]	Bag of words	SVM	91.28%
Cheng et al. [19]	Local features using Fisher Vector	SVM	94.68%
Abir et al. [20]	GLCM	PNN	83.33%
Deepak and Ameer [21]	GoogleNet	SVM	97.10%
Afshar et al. [22]	Capsule networks (CapsNet)	−	86.56%
Swati et al. [23]	Fine-tune VGG19	−	94.80%
Arı et al. [24]	AlexNet and VGG16	ELM	97.64%
Kaplan et al. [11]	nLBP ve $\alpha$LBP	KNN	95.56%
Belaid and Loudini [25]	VGG16	Softmax	96.5%
Kaur and Gandhi [26]	Fine-tuned AlexNet	−	96.95%
Rehman et al. [27]	VGG16	Softmax	98.69%
Deepak and Ameer [6]	CNN	SVM	95.82%
Bodapati et al. [28]	Xception and InceptionResNetV2	Softmax	95.23%
Sadad et al. [29]	NASNet	Softmax	99.6%
Oksuz et al. [30]	ResNet18+ShallowNet	SVM	97.25%
Ayadi et al. [31]	DSURF and HoG	SVM	90.27%
MTAP model	CNN	Softmax	99.69%

**Fig. 7:**
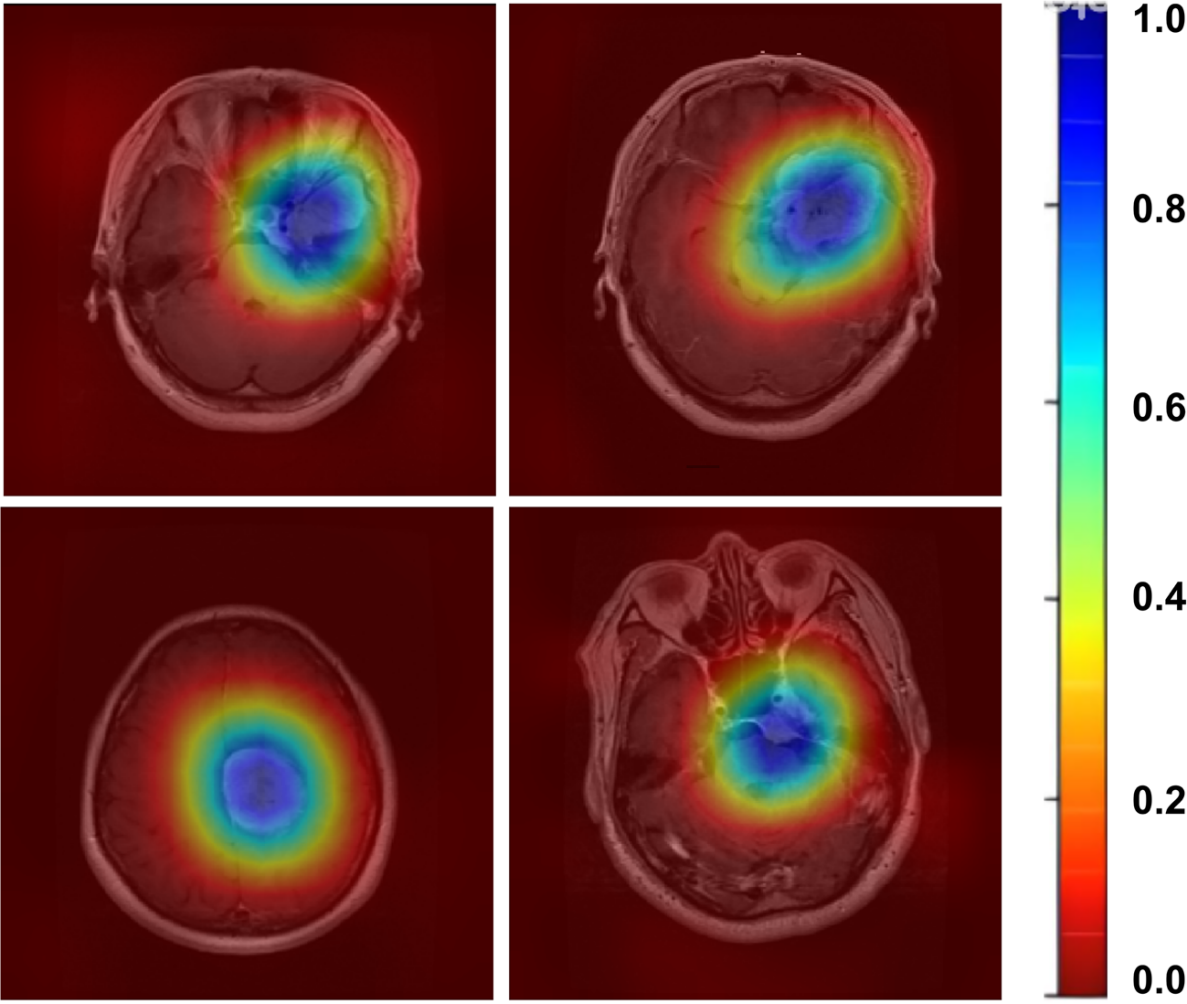
Grad-CAM heat map visualization results

We implemented a non-local block [32] attention mechanism in our proposed model to enhance its ability to focus on relevant image sections for more accurate tumor classification. Additionally, we tested advanced regularization techniques such as SpatialDropout and AlphaDropout variants to reduce overfitting and improve generalization capabilities. However, we observed that these methods did not yield a significant improvement in the performance of our final model. These results suggest that the effectiveness of specific techniques and mechanisms can vary depending on the dataset used, model architecture, and the specific problem context. Therefore, the integration of these techniques into our model was not carried out, as they did not lead to the anticipated performance enhancement.

A pie chart depicting the ratios of correct and incorrect predictions for each class was created to visually represent categorization performance across multiple classes. The graphic in Fig. 6 shows the model’s prediction accuracy for each class, with each slice representing a different class. The percentage labels on the graphic represent the proportion of correct predictions compared to the total samples for each class.

The comparative analysis of the results obtained in this study with the findings reported in previous studies conducted on the same dataset is presented in Table 10. This tabulated comparison provides valuable insights into the performance of our proposed model, i.e., MTAP, in the context of brain tumor classification. We hope to situate the breakthroughs and contributions of our study within the broader landscape of brain tumor detection approaches by comparing our findings with established literature.

Brain tumor classification studies conducted on the Figshare dataset reveal that, when examining the performances presented in Table 10, our proposed MTAP model provides state-of-the-art results with an accuracy of 99.69%. To enhance the interpretability of our proposed method in brain tumor classification on the Figshare dataset, we employed heat map visualization methods. The primary objective was to elucidate the spatial attention patterns of the model across input images, aiming to identify regions of heightened attention within brain tumor images during the classification process. Heat map visualization proves to be a potent tool for comprehending the dynamic attentional mechanism of the model, revealing relevant features and regions that significantly influence the model’s classification decisions. The utilization of this approach markedly improves the interpretability of our proposed method, offering valuable insights into the model’s underlying structure concerning brain tumor classification. As depicted in Fig. 7, the parietal and temporal lobes emerge as focal points in the model’s attention allocation patterns, underscoring their importance in the analysis of the resulting heat map. This finding suggests the model’s nuanced detection of distinct characteristics or patterns highly indicative of different tumor types, particularly within the parietal and temporal lobes.

## Discussion

In this paper, we provide a detailed analysis and improvement in the domain of brain tumor classification using deep learning methods. Our major goal was to improve the accuracy and dependability of brain tumor classification, which is a critical component of medical diagnostics. Through a systematic and thorough method, we introduce the MTAP model, a new neural network architecture that integrates model pruning, ADASYN to deal with class imbalance, and Avg-TopK pooling for enhanced feature extraction. The results are highly promising, demonstrating state-of-the-art accuracy at 99.69%.

Examination of the heat maps generated by the model reveals a predominant focus on the parietal and temporal lobes. This suggests the model effectively identifies distinctive features within the parietal and temporal lobes, crucial for classifying tumor types.

The MTAP model represents the potential power of using artificial intelligence in brain tumor classification. The proposed approach supports integration and advancement potential for similar problem domains. Our study makes a positive contribution to the broader exploration of artificial intelligence applications in improving healthcare services, particularly in the context of brain tumor classification. In the continuation of this work, the performance of the proposed model will be investigated on different datasets.
